# Biographic Clinics

**Published:** 1906-05

**Authors:** 


### BOOKS AND AUTHORS.

**Biographic Clinics.** Essays concerning the Influence of Visual Function Pathologic and Physiologic Upon the Health of Patients. By **George M. Gould,** M.D., Editor of American Medicine. Volume III. 12mo., 516 pages. Philadelphia: P. Blakiston's Son and Co. 1905. (Price, $1.00).

This volume is the third of the series of Biographic Clinics, in which Gould has portrayed the miseries of distinguished personages, which he believes, in the light of present-day experiences, might have been relieved by optical therapy. The clinics hereby included are those of John Addington Symonds and of Taine. The rest of the book, and also by far the larger part of it, is made up of papers and addresses written and published at different times by the author, giving his views on the new ophthalmology, optic and ocular factors in the etiology of the scoliosis of school children, visual function the cause of slanted handwriting; its relation to school hygiene, school desks, malposture, spinal curvature, and myopia, dextrality and sinistrality, the pathologic results of dextrocularity and sinistrocularity, subnormal accommodation, some problems of presbyopia, besides papers by himself on the history and etiology of migraine, and on the reception of medical discoveries, together with one by Simeon Snell, of Sheffield, England, on eyestrain as a cause of headache and other neuroses, and one by C. Ernest Pronger, of slight errors of refraction and their influence on the nervous system. Two appendices are added on suggestions as to postmydriatic refraction tests, and a case of mathematically perfect eyes.

This collection of miscellaneous matter contains many facts of impressive significance, and worthy of thoughtful consideration. The reader may not accept some of the conclusions of the author,—they are at times rather radical,—neither will he fully sympathise with the author's impatience, not to use a stronger term, with ophthalmologists, who, for cogent reasons, have differed with him. It is to be hoped that Gould's views will receive due and respectful attention; but it should not, on the other hand, be forgotten that there are others who are equally entitled to our consideration, but who disagree with him on some points most decidedly.

